# Discovery and validation of autosomal dominant Alzheimer’s disease mutations

**DOI:** 10.1186/s13195-018-0392-9

**Published:** 2018-07-18

**Authors:** Simon Hsu, Brian A. Gordon, Russ Hornbeck, Joanne B. Norton, Denise Levitch, Adia Louden, Ellen Ziegemeier, Robert Laforce, Jasmeer Chhatwal, Gregory S. Day, Eric McDade, John C. Morris, Anne M. Fagan, Tammie L. S. Benzinger, Alison M. Goate, Carlos Cruchaga, Randall J. Bateman, Dominantly Inherited Alzheimer Network (DIAN), Celeste M. Karch

**Affiliations:** 1 Department of Psychiatry, Washington University School of Medicine, 425 S. Euclid Ave, Campus Box 8134, St. Louis, MO 63110 USA; 2 Department of Radiology, Washington University School of Medicine, St. Louis, MO USA; 3 Department of Neurology, Washington University School of Medicine, St. Louis, MO USA; 4 Clinique Interdisciplinaire de Mémoire du CHU de Québec, Département des Sciences Neurologiques, Faculté de Médecine, Université Laval, Québec City, Québec Canada; 5 Massachusetts General Hospital/Martinos Center for Biomedical Imaging, 149 13th Street, Gerontology Research Room 2669, Charlestown, MA 02129 USA; 6 Mallinckrodt Institute of Radiology, Washington University School of Medicine, St. Louis, MO 63110 USA; 7 Department of Neuroscience, Mount Sinai School of Medicine, New York, NY USA

**Keywords:** APP, PSEN1, PSEN2, Autosomal dominant Alzheimer’s disease, Cell-based assays, Pathogenicity algorithm

## Abstract

**Background:**

Alzheimer’s disease (AD) is a neurodegenerative disease that is clinically characterized by progressive cognitive decline. Mutations in amyloid-β precursor protein (*APP*), presenilin 1 (*PSEN1*), and presenilin 2 (PSEN2) are the pathogenic cause of autosomal dominant AD (ADAD). However, polymorphisms also exist within these genes.

**Methods:**

In order to distinguish polymorphisms from pathogenic mutations, the DIAN Expanded Registry has implemented an algorithm for determining ADAD pathogenicity using available information from multiple domains, including genetic, bioinformatic, clinical, imaging, and biofluid measures and in vitro analyses.

**Results:**

We propose that *PSEN1* M84V, *PSEN1* A396T, *PSEN2* R284G, and *APP* T719N are likely pathogenic mutations, whereas *PSEN1* c.379_382delXXXXinsG and *PSEN2* L238F have uncertain pathogenicity.

**Conclusions:**

In defining a subset of these variants as pathogenic, individuals from these families can now be enrolled in observational and clinical trials. This study outlines a critical approach for translating genetic data into meaningful clinical outcomes.

## Background

Alzheimer’s disease (AD) is characterized clinically by progressive cognitive decline and neuropathologically by progressive neuronal loss and the accumulation of amyloid plaques and neurofibrillary tangles. Mutations in amyloid-β precursor protein (*APP*), presenilin 1 (*PSEN1*) and presenilin 2 (*PSEN2*) are the pathogenic cause of autosomal dominant AD (ADAD). More than 200 pathogenic mutations have been identified in *APP*, *PSEN1*, and *PSEN2* (reviewed in [1, 2]). PSEN1 and PSEN2 form the catalytic domain of the γ-secretase complex, which is involved in sequential cleavage of APP into amyloid-β (Aβ) peptides.

The Dominantly Inherited Alzheimer Network (DIAN) is an observational study designed to follow families with mutations in *APP*, *PSEN1*, and *PSEN2* that cause ADAD [3]. The DIAN Expanded Registry (DIAN EXR; www.dianexr.org) is a web-based registry with global outreach to ADAD families and investigators. It functions to identify families with ADAD and to determine the causative mutations through a genetic discovery program. This program includes genetic counseling and testing for known and unknown causes of ADAD. During a period of 6 years (2011 to 2017), the DIAN Clinical/Genetics committee reviewed 150 pedigrees, 76 of which were approved for genetic counseling and testing. Forty-six probands were positive for a coding variant in *APP*, *PSEN1*, or *PSEN2*, including 39 individuals with known pathogenic mutations, 6 with variants of unknown pathogenic significance, and 1 variant that was determined to be an AD risk factor.

In some cases, families are identified with several generations of early-onset AD; however, at the time of enrollment, whether a pathogenic mutation is the cause of disease in these families remains unknown. We performed genetic analyses in research participants from six densely affected early-onset AD pedigrees and identified variants in *APP*, *PSEN1*, and *PSEN2* with unknown pathogenicity. In all families examined, we lacked sufficient genetic material to perform segregation analyses (e.g., DNA was available from only one or two family members). To assess the pathogenicity of novel variants in *APP*, *PSEN1*, and *PSEN2* when pedigree and clinical data are limited or incomplete, Guerreiro and colleagues [4] proposed a pathogenicity algorithm. In the present study, we modified and expanded this algorithm to evaluate the pathogenicity of these six variants using genetic, biochemical, biomarker, and clinical data.

## Methods

### Genetic screening

Genomic DNA was extracted from peripheral blood lymphocytes using standard protocols. All coding exons in *APP*, *PSEN1*, and/or *PSEN2* were amplified and sequenced using the BigDye Terminator version 3.1 cycle sequencing kit (Life Technologies, Carlsbad, CA, USA) and analyzed on an ABI 3500 genetic analyzer (Life Technologies). Sequence analysis was performed using Sequencher software (Gene Codes, Ann Arbor, MI, USA).

### Bioinformatics

To determine whether *APP*, *PSEN1*, and *PSEN2* variants represented rare or common polymorphisms, we investigated two population-based exome sequencing databases: the Exome Variant Server (EVS) and Exome Aggregation Consortium (ExAC) browser. Polymorphism phenotype v2 (PolyPhen-2; [5]) and Sorting Intolerant From Tolerant (SIFT) were used to predict whether the amino acid change would be disruptive to the encoded protein.

### Cerebrospinal fluid

Cerebrospinal fluid (CSF) was collected by lumbar puncture under fasting conditions from the *PSEN1* M84V carrier and noncarrier. CSF Aβ_42_, total tau, and tau phosphorylated at threonine 181 (p-tau_181_) were measured by immunoassay (xMAP; Luminex, Austin, TX, USA) as previously described [6].

### PiB imaging

Structural magnetic resonance imaging (MRI) was performed using 3-T scanners and following the Alzheimer’s Disease Neuroimaging Initiative (ADNI) protocol [7, 8]. T1-weighted scans were processed through FreeSurfer. Positron emission tomography with Pittsburgh compound B (PiB) was coregistered with MRI images. The standardized uptake value ratio (SUVR) was calculated for each region [9, 10]. Global burden was characterized using a summary measure [9]. These data were collected for a *PSEN1* M84V carrier and noncarrier.

### Biochemical analysis

#### Plasmids and mutagenesis

The full-length *PSEN1* complementary DNA (cDNA) was cloned into pcDNA3.1 Myc/His vector [11]. The M84V, c.379_382delXXXXinsG, or A396T variant was introduced into the *PSEN1* cDNA using the QuikChange II XL Site-Directed Mutagenesis Kit (Agilent Technologies, Santa Clara, CA, USA). Clones were sequenced to confirm the presence of the variant and the absence of additional modifications. *PSEN1* wild type (WT) and the pathogenic *PSEN1* A79V mutation were included as controls.

The full-length *PSEN2* cDNA was cloned into pcDNA3.1 vector [12]. The L238F or R284G variant was introduced into the *PSEN2* cDNA and screened as described above. *PSEN2* WT and the pathogenic *PSEN2* N141I mutation were included as controls [13].

The full-length *APP* cDNA (isoform 695) was cloned into pcDNA3.1 [14]. The T719N variant was introduced into the APP cDNA and screened as described above. APP WT and the pathogenic APP KM670/671NL(Swe) mutation were included as controls.

#### Transient transfection

To assess novel *PSEN1* and *PSEN2* variants, we used neuroblastoma cells (N2A) stably expressing human APP WT (695 isoform; N2A695). To assess novel *APP* variants, we used N2A. N2A cells were maintained in equal amounts of DMEM and Opti-MEM, supplemented with 5% FBS, 2 mM L-glutamine, 100 μg/ml penicillin/streptomycin, and, for stable cells, 200 μg/ml G418 (Life Technologies). Upon reaching confluence, cells were transiently transfected with Lipofectamine 2000 reagent (Life Technologies). Culture media were replaced after 24 hours, and cells were incubated for another 24 hours prior to analysis. Four independent transfections were performed for each construct and used for subsequent analyses.

#### Aβ enzyme-linked immunosorbent assay

Conditioned medium was collected and centrifuged at 3000 × *g* at 4 °C for 10 minutes to remove cell debris. The levels of Aβ_40_ and Aβ_42_ were measured in cell culture media by sandwich enzyme-linked immunosorbent assay (ELISA) as described by the manufacturer (Life Technologies). To account for variability in transfection efficiency between experiments, ELISA values were obtained (pg/ml) and corrected for total intracellular protein (μg/ml). Statistical difference was measured using an unpaired Student’s *t* test.

## Results and discussion

### PSEN1 M84V

**Fig. 1:**
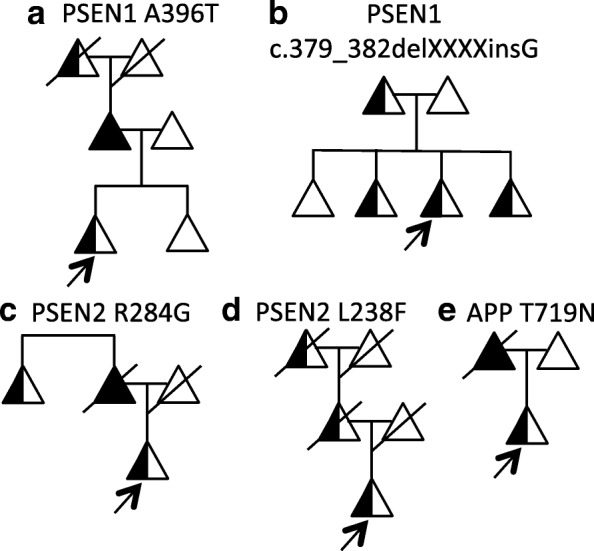
Identification of *APP*, *PSEN1*, and *PSEN2* variants in densely affected Alzheimer’s disease (AD) pedigrees. **a–e** Pedigrees. *Half-shaded triangles* represent individuals with a clinical diagnosis of symptomatic AD. *Fully shaded triangles* represent individuals with autopsy-confirmed symptomatic AD. *Diagonal lines* represent deceased individuals. *Arrows* indicate those individuals with DNA, all of whom are mutation/variant carriers. Pedigrees have been masked to maintain anonymity. The pedigree of the *PSEN1* M84V family was excluded to prevent potential disclosure of mutation status in asymptomatic mutation carriers

The proband was identified in a family with three generations of early-onset AD and with a mean age at onset of 59 years (Fig. 1; pedigree not shown to avoid disclosing mutation status to asymptomatic proband and other family members). Sequencing of the proband revealed a single base substitution (ATG to GTG) at codon 84 in exon 4 of *PSEN1*, resulting in a methionine-to-valine change (M84V). *PSEN1* M84V was also identified in a family-based sequencing study of AD (National Institute of Mental Health Alzheimer’s Disease Genetics Initiative Study) [15]. Two *PSEN1* M84V carriers were confirmed to have had AD at autopsy with ages at symptomatic onset of 70 and 72 years. The third carrier was cognitively normal at age 67 years [15].

To determine whether *PSEN1* M84V represents a rare polymorphism, we examined two population-based exome sequencing databases (Table 1). *PSEN1* M84V was absent from both EVS and ExAC. *PSEN1* M84L was identified in one individual in the ExAC browser. *PSEN1* M84V was also absent in more than 1700 AD and control samples with whole-exome sequencing.

**Table 1:** Bioinformatic analysis of *APP*, *PSEN1*, and *PSEN2* variants of unknown pathogenicity

Gene	Variant	EVS^a^	ExAC^b^	PolyPhen	SIFT	Variant previously reported	Location	PSEN1-PSEN2 conservation
*APP*	T719N	0	0	Probably damaging	Damaging	Yes	Exon 17	N/A
*PSEN1*	M84V	0	0	Probably damaging	Tolerated	Yes	Exon 4 (TM-1)	Yes
*PSEN1*	c.379_382delXXXXinsG	0	0	N/A	Tolerated	No	Exon 5 (HL1)	Yes
*PSEN1*	A396T	0	0	Probably damaging	Tolerated	Yes	Exon 11 (HL8)	No
*PSEN2*	L238F	2	2	Probably damaging	Damaging	Yes	Exon 7 (TM-V)	Yes
*PSEN2*	R284G	0	0	Probably damaging	Damaging	No	Exon 8	Yes

*Abbreviations: PolyPhen* Polymorphism phenotype, *SIFT* Sorting Intolerant From Tolerant, *EVS* Exome Variant Server, *ExAC* Exome Aggregation Consortium, *PSEN* Presenilin, *APP* Amyloid precursor protein

^a^Represents sequence data from 4300 unrelated European Americans (8598 alleles)

^b^Represents sequence data from 60,706 unrelated European Americans (121,204 alleles)

**Fig. 2:**
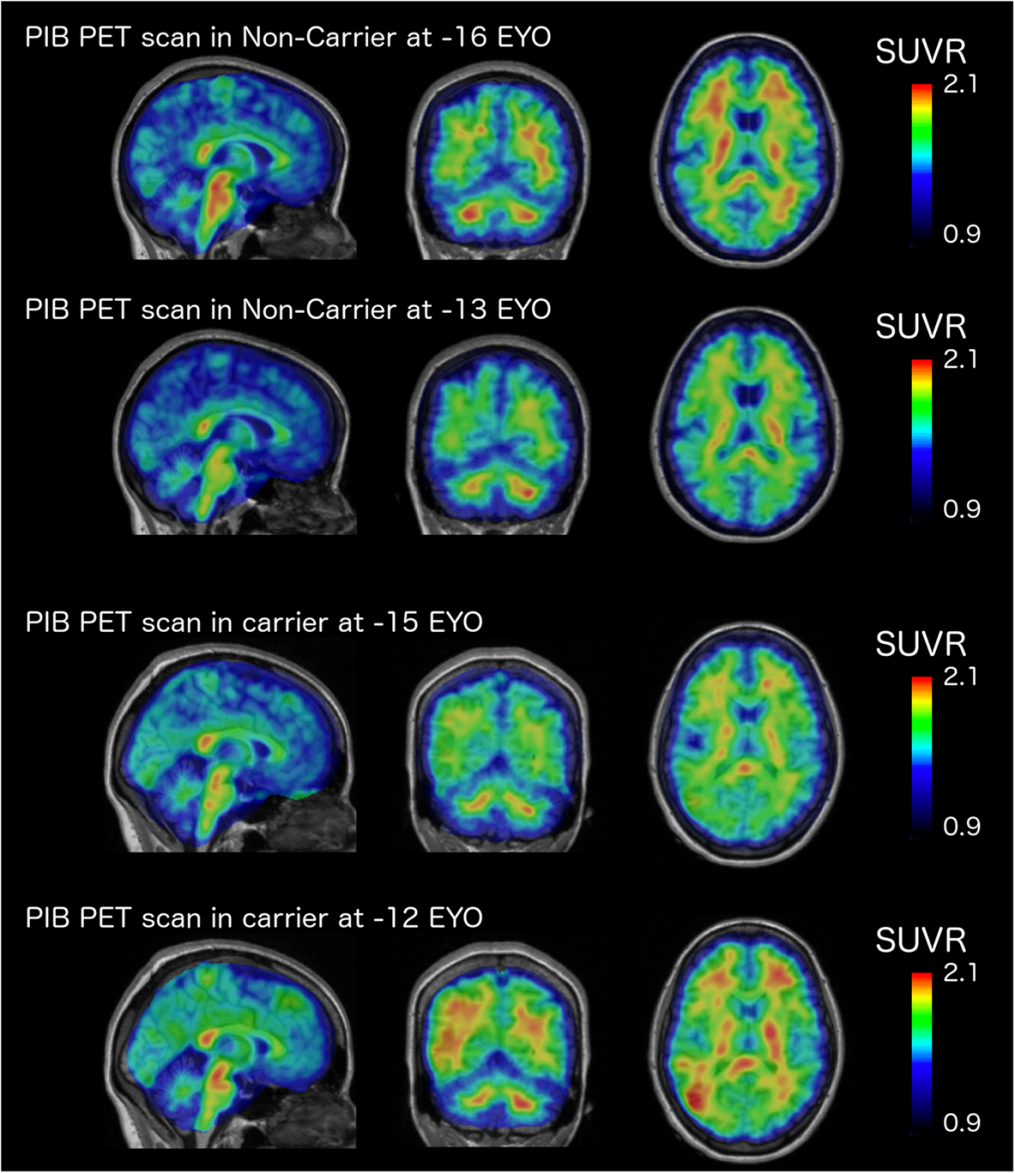
Pittsburgh compound B (PiB) uptake in the brain of a presymptomatic *PSEN1* M84V carrier is consistent with presymptomatic autosomal dominant Alzheimer’s disease mutation carriers. ^11^C-PiB positron emission tomographic scans were performed longitudinally in a *PSEN1* M84V noncarrier and carrier. The color scale for standardized uptake values (SUV) indicate red (high), yellow (medium), and blue (low) PiB retention. *EYO* Estimated years of onset

CSF Aβ, total tau, and phosphorylated tau were measured in the *PSEN1* M84V carrier, who was cognitively normal at the time of lumbar puncture. CSF Aβ (361.5 pg/ml), total tau (84.93 pg/ml), and p-tau_181_ (22.02 pg/ml) at 11 years prior to the parental age at symptomatic onset was consistent with other presymptomatic pathogenic mutation carriers but not completely distinct from noncarriers [3, 6]. The *PSEN1* M84V carrier produced a mean cortical PiB SUVR value of 1.132 at 15 years prior to the parental age at symptomatic onset and 1.209 at 11 years prior to parent age of disease onset (Fig. 2). A related noncarrier was imaged at 16 years prior to the parent age of disease onset and produced mean cortical PiB SUVR values of 1.086 and 1.039 at 13 years prior to age at disease onset in the parent (Fig. 2). Thus, fluid and imaging biomarkers were consistent with those observed in presymptomatic ADAD mutation carriers.

**Fig. 3:**
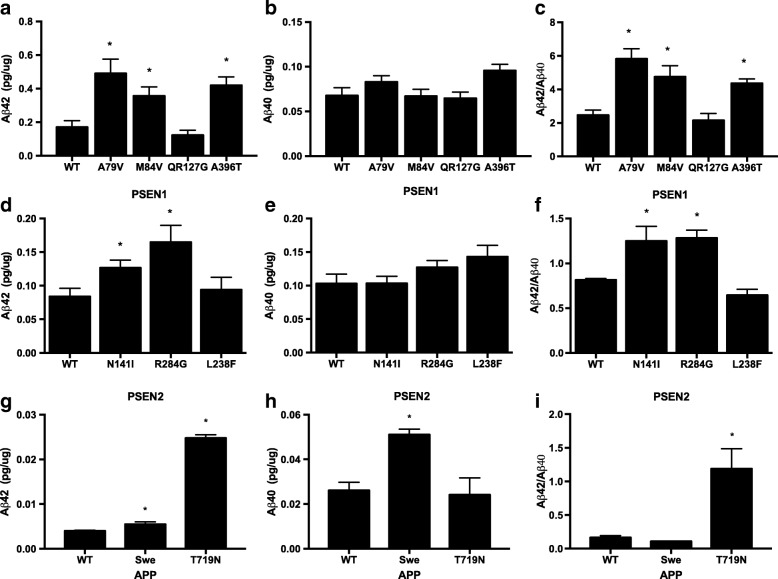
Amyloid-β 1–42 peptide (Aβ_42_) and Aβ_40_ in cells expressing *APP*, *PSEN1*, and *PSEN2* variants of unknown pathogenicity. N2A695 cells were transfected with vectors expressing presenilin 1 or 2. Media was replaced 24 hours posttransfection and incubated for an additional 24 hours. Media were collected, and Aβ_42_ and Aβ_40_ were measured by enzyme-linked immunosorbent assay (ELISA) (pg/ml). Total intracellular protein was measured by bicinchoninic acid assay and used to normalize to ELISA Aβ values, resulting in a value represented as pg/μg (*see* the Methods section of text). **a–c** PSEN1 wild type (WT), pathogenic mutation A79V, and variants with unknown pathogenicity. **a** Aβ_42_. **b** Aβ_40_. **c** Aβ_42_/Aβ_40_ ratio. **d–f** PSEN2 WT, pathogenic mutation N141I, and variants with unknown pathogenicity. **d** Aβ_42_. **e** Aβ_40_. **f** Aβ_42_/Aβ_40_ ratio. **g–i** APP WT, pathogenic mutation KM670/671NL(Swe), and APP T719N. Graphs represent the mean (±SEM) of four replicate experiments. * *p* < 0.05. *PSEN1* QR127G is the amino acid representation for *PSEN1* c.379_382delXXXXinsG

We next sought to determine whether the *PSEN1* M84V variant alters Aβ isoform levels in a manner consistent with previously reported pathogenic *PSEN1* mutations. We expressed vectors containing *PSEN1* WT, A79V (a known pathogenic mutation), and M84V in N2A695 cells. We found that cells expressing *PSEN1* M84V produced significantly more Aβ_42_ than cells expressing *PSEN1* WT (Fig. 3a–c). We also observed a significant increase in the Aβ_42_/Aβ_40_ ratio in cells expressing *PSEN1* M84V or the pathogenic *PSEN1* A79V- compared with *PSEN1* WT-expressing cells (Fig. 3c).

**Fig. 4:**
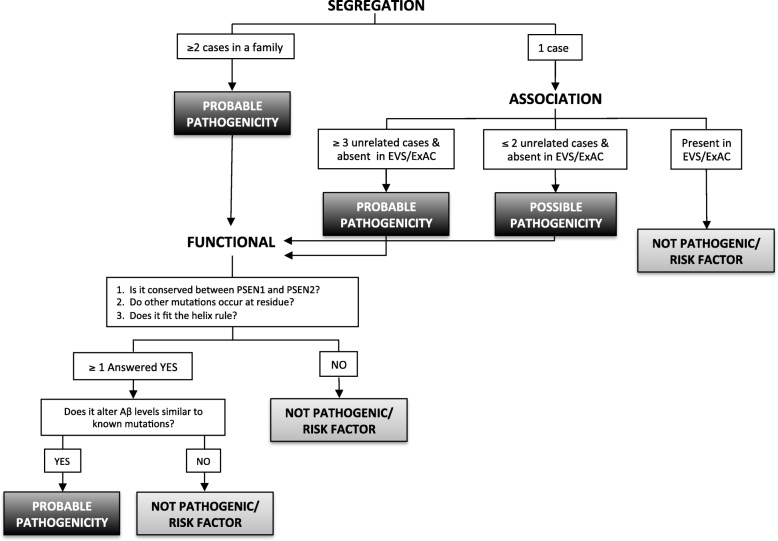
Algorithm to classify the benign or pathogenic nature of *APP*, *PSEN1*, and *PSEN2* variants. This model is modified from the algorithm previously proposed by Guerreiro et al. in 2010 [4]. The modifications include the evaluation of variants in the Exome Variant Server and Exome Aggregation Consortium databases and a tiered approach to evaluating functional studies that more heavily weighs the impact of the variant on amyloid-β 1–42 peptide (Aβ_42_) and Aβ_40_ levels on pathogenicity

Thus, we can apply a pathogenicity algorithm, modified from Guerreiro and colleagues [4], to assess the pathogenicity of *PSEN1* M84V (Fig. 4). *PSEN1* M84V occurs within exon 4 and the first transmembrane domain. This residue is highly conserved between *PSEN1* and *PSEN2* [16]. Additionally, a pathogenic mutation has been reported at this site: ΔI83/M84 [17, 18]. In a cell model, Aβ levels were consistent with pathogenic mutations (Fig. 3a–c, g). Thus, *PSEN1* M84V satisfies all criteria for pathogenicity based on residue and Aβ levels [4]. Taken together, we propose that *PSEN1* M84V represents a pathogenic mutation.

### PSEN1 A396T

The proband was identified in a family with three generations of early-onset AD (Fig. 1a). The proband had an age at symptomatic onset of 50 years. The parent of the proband had an age at symptomatic onset of 57 years, with AD confirmed at autopsy at age 67 years. Sequencing of the proband (Fig. 1a) revealed a single base substitution (GCG to ACG) at codon 396 in exon 11 of *PSEN1*, resulting in an alanine-to-threonine change (A396T). This variant was reported previously in one individual with sporadic AD [19]. *PSEN1* A396T was absent in the EVS and ExAC databases (Table 1). We found that cells expressing *PSEN1* A396T produced significantly more Aβ_42_ than cells expressing *PSEN1* WT (Fig. 3a–c). Thus, applying the algorithm for assessing pathogenicity (Fig. 4), we propose that the *PSEN1* A396T represents a probable pathogenic mutation.

### PSEN1 c.379_382delXXXXinsG

The proband had an age at symptomatic onset of 50 years, with an average age at symptomatic onset in family members aged 43 years (Fig. 1b). Sequencing of the proband (Fig. 1b) revealed a 4-bp deletion and insertion of G at codon 127 in exon 5 of *PSEN1*, resulting in the deletion of glutamine and arginine and insertion of glycine (c.379_382delXXXXinsG). This is a novel variant and was absent from the EVS and ExAC databases (Table 1). N2A695 expressing *PSEN1* c.379_382delXXXXinsG produced Aβ_42_ and Aβ_40_ levels similar to *PSEN1* WT (Fig. 3a–c). Thus, we propose that *PSEN1* c.379_382delXXXXinsG is an AD risk factor or benign polymorphism.

### PSEN2 R284G

The proband had an age at symptomatic onset of 58 years, with average age at symptomatic onset in the family of 56 years (Fig. 1c). Sequencing of the proband (Fig. 1c) revealed a single base pair substitution (CGG to GGG) at codon 284 in exon 8 of *PSEN2*, resulting in an arginine-to-glycine change (R284G). This is a novel variant and was absent from the EVS and ExAC databases (Table 1).

To assess the effects of *PSEN2* variants on Aβ isoform levels, we expressed vectors containing *PSEN2* WT, N141I, and R284G in N2A695 cells. Cells expressing *PSEN2* N141I and R284G produced significantly more Aβ_42_ than cells expressing *PSEN2* WT (Fig. 3d and e). The Aβ_42_/Aβ_40_ ratio was also significantly higher in cells expressing *PSEN2* N141I and R284G (Fig. 3f). Thus, applying the algorithm for assessing pathogenicity (Fig. 4), we propose that the *PSEN2* R284G represents a probable pathogenic mutation.

### PSEN2 L238F

The proband was identified in a family with three generations of early-onset AD (Fig. 1d). The proband had an age at symptomatic onset of 49. The parent of the proband was diagnosed with AD at 57 years of age and died at age 77. Sequencing of the proband (Fig. 1d) revealed a single base pair substitution (CTT to TTT) at codon 238 in exon 7 of *PSEN2*, resulting in a leucine-to-phenylalanine change (L238F). *PSEN2* L238F was also identified in two alleles in EVS, in two alleles in ExAC, and in one individual with sporadic AD (Table 1; [20]). CSF Aβ, total tau, and phosphorylated tau were measured in a *PSEN2* L238F carrier related to the proband, who was cognitively normal at the time of lumbar puncture. CSF Aβ (506.54 pg/ml), total tau (38.75 pg/ml), and p-tau_181_ (21.33 pg/ml) at 17 years prior to the parental age at symptomatic onset was consistent with biomarker levels in normal controls [3, 6]. Cells expressing *PSEN2* L238F produced Aβ_42_ and Aβ_40_ levels similar to *PSEN2* WT (Fig. 3d–f). Thus, we propose that *PSEN2* L238F represents an AD risk factor or benign polymorphism.

### App T719N

The proband was identified in a family with two generations of early-onset AD (Fig. 1e). The proband had an age at symptomatic onset of 45 years. The parent of the proband was diagnosed at 45 years of age. Sequencing of the proband (Fig. 1e) revealed a single base pair substitution (ACC to AAC) at codon 719 in exon 17 of *APP*, resulting in a threonine-to-asparagine change (T719N). *APP* T719N was absent in the EVS and ExAC databases and was detected in one individual with early-onset AD (Table 1) [21]. Cells expressing *APP* T719N produced significantly elevated levels of Aβ_42_ and Aβ_42_/Aβ_40_ relative to *APP* WT (Fig. 3g–i ). Thus, we propose that *APP* T719N is a probable pathogenic mutation.

## Conclusions

By applying genetic, bioinformatic, and functional data to an algorithm to assess pathogenicity, we propose that the *PSEN1* M84V, *PSEN1* A396T, *PSEN2* R284G, and *APP* T719N are likely pathogenic mutations resulting in ADAD, whereas *PSEN1* c.379_382delXXXXinsG and *PSEN2* L238F are likely benign polymorphisms. This algorithm was adapted and modified from a pathogenicity algorithm originally proposed by Guerreiro and colleagues [4]. We have expanded upon this algorithm in several important ways: (1) expanding the number of controls in the association analyses from 100 to 65,000 by exploiting the EVS and ExAC databases; (2) evaluating the bioinformatic functional findings (e.g., conservation between *PSEN1* and *PSEN2* and the presence of other mutations at the same residue) independent of the cell-based functional findings; and (3) incorporating cell-based assays to evaluate the impact of novel variants on Aβ levels. We propose that this modified approach to assessing pathogenicity provides an important pipeline for incorporating mutation data at several levels and that this algorithm may be adapted to impute pathogenicity when extensive genetic data are missing for affected families. Designation of a variant as pathogenic will allow individuals to enroll in observational and clinical trials for AD, with clear applications in clinical and research settings.
